# Combined Uyghur medicine, acupuncture, and rehabilitation for traumatic oculomotor nerve palsy: A case report

**DOI:** 10.1097/MD.0000000000047006

**Published:** 2026-01-23

**Authors:** Ailiyaer Yasheng, Gulinishahan Abulimiti, Nilupaer Xiefukaiti, Yang Liu, Gulinazi Rouzi, Aerziguli Aizezi, Zehuai Wen, Aizezi Aihemaitiniyazi

**Affiliations:** a Department of Rehabilitation, Uyghur Medical Hospital of Xinjiang Uyghur Autonomous Region, Urumqi, China; b Key Laboratory of Evidence-Based and Translation, Xinjiang Hospital Preparation of Traditional Chinese Medicine, Urumqi, China; c Department of Neurology, Second Affiliated Hospital of Xinjiang Medical University, Urumqi, China; d Center for Clinical Research, Guangdong Provincial Hospital of Chinese Medicine,Guangzhou University of Chinese Medicine, Guangzhou, China.

**Keywords:** acupuncture therapy, case report, rehabilitation training, traumatic oculomotor nerve palsy, Uyghur medicine

## Abstract

**Background:**

Oculomotor nerve palsy caused by craniocerebral injury often leads to a significant decline in patients’ quality of life. This study aims to explore a comprehensive treatment approach combining Uyghur medicine, rehabilitation training, and acupuncture therapy, and to present a case report evaluating its clinical efficacy in improving ocular motor function and related symptoms.

**Case presentation:**

A patient sustained craniocerebral injury due to a traffic accident and presented with severe oculomotor nerve palsy. Initial head CT scan revealed multiple intracranial injuries.

**Interventions:**

The patient received comprehensive treatment consisting of Uyghur medicine guided by differential diagnosis, acupuncture therapy, and structured ocular motor rehabilitation training. The rehabilitation training was administered by a certified rehabilitation therapist with an intermediate professional title and included: eye movement exercises (10–15 times/day), eyelid lifting training (10–20 times/day), visual rehabilitation training (10–15 times/day), and pupillary response training (2–3 times/day), all conducted over a 20-day period. Clinical outcomes, including palpebral fissure height, diplopia score, and pupillary function, were systematically evaluated by the rehabilitation therapist at admission, on day 10, on day 24 (pre-discharge). The overall quality of life of patients was assessed using the 36-item Short Form Health Survey. The study was approved by the institutional ethics committee, and written informed consent was obtained from the patient.

**Results:**

Following comprehensive treatment, the patient demonstrated sustained improvement. The 10-day assessment revealed complete resolution of left ptosis and exotropia. Only mild diplopia persisted during esophoria, with moderate improvement in pupillary asymmetry (approximately 1 mm difference). The pre-discharge assessment at 24 days confirmed complete resolution of all primary symptoms (including ptosis, strabismus, and diplopia; diplopia score: 0). Pupil size, shape, and light reflexes returned to normal. No symptom recurrence was observed at the 3-month follow-up; CT imaging demonstrated complete hematoma absorption, fracture healing, and bone remodeling. The patient’s 36-item Short Form Health Survey quality of life assessment showed significant improvement across all dimensions.

**Conclusion:**

This case demonstrates that an integrated treatment approach combining Uyghur medicine, rehabilitation training, and acupuncture yields significant therapeutic efficacy in improving ocular motor dysfunction secondary to complex traumatic brain injury.

## 1. Introduction

Oculomotor nerve palsy (ONP), also known as 3rd cranial nerve palsy, is a neuro-ophthalmic syndrome caused by oculomotor nerve dysfunction or injury.^[1]^ Clinical manifestations include ptosis, diplopia, pupillary dilation, and impaired medial and superior gaze.^[2]^ The incidence of this condition increases with age, peaking in the 70-year-old and 90-year-old age groups, with an incidence rate of 3.7 to 4.0 cases per 100,000 population.^[3]^ Research demonstrates that trauma is a critical factor in the etiology of ONP, accounting for up to 26% of cases.^[4,5]^ The probability of complete recovery from traumatic ONP is approximately 0.5%. Associated craniocerebral injuries may result in irreversible nerve damage, causing permanent functional impairment and significantly reducing quality of life.^[6]^ Surgical intervention is the primary treatment modality for patients with pupil-involving ONP and congenital palsy unresponsive to conservative management for more than 6 months.^[7]^ Shingai Y^[8]^ successfully treated ocular neuromyotonia secondary to recurrent sphenoid ridge meningioma through tumor resection to alleviate oculomotor nerve compression. Tabata Shinya^[9]^ achieved favorable therapeutic outcomes using surgical clipping or endovascular embolization for patients with ONP caused by unruptured posterior communicating artery aneurysms.

Non-surgical treatment research primarily focuses on etiological intervention and nerve function restoration during the acute phase (within 6 months of onset). Tremblay C^[10]^ reported a case of a 40-year-old male with partial ONP who achieved complete recovery within 2 months following dexamethasone therapy. Inchara N^[11]^ conducted a controlled study of 50 patients with cranial nerve palsies, demonstrating significant therapeutic efficacy in patients receiving vitamin B12 injections. In China, traditional Chinese medicine (TCM) functions as a parallel system to modern medicine.^[12]^ According to TCM theory, treatment strategies frequently involve “tonifying qi and activating blood circulation to remove stasis” or “dispelling wind and dredging collaterals.” Luo D^[13]^ formulated a proprietary herbal prescription containing peach kernel, safflower, and astragalus, which yielded favorable therapeutic outcomes. Acupuncture represents a core therapeutic modality in TCM, encompassing traditional acupuncture, electroacupuncture, wheat grain moxibustion, and ocular acupuncture techniques. For instance, Chen Jian^[14]^ employed the “awakening brain and opening orifices” acupuncture method, while Zhang^[15]^ utilized Zheng acupuncture technique, both demonstrating effective improvement of clinical symptoms. Uyghur medicine constitutes one of the ethnic theoretical systems within Chinese medicine.^[16]^ While TCM broadly emphasizes achieving systemic balance through herbal synergistic effects and energy flow regulation, Uyghur medicine focuses on ecological harmony and personalized humor regulation.

This study presents a case of ONP resulting from traumatic brain injury (TBI), treated with a 3-dimensional integrated therapy combining traditional Uyghur medicine, acupuncture, and modern rehabilitation training. This integrated protocol has been successfully implemented in clinical practice, achieving satisfactory therapeutic outcomes within a short period, though it has not been previously documented in academic literature. This case report differs from prior studies that predominantly focused on either modern medical interventions, TCM monotherapy, or dual therapeutic approaches. The aim of this research is to systematically review the treatment process and share clinical experience, potentially establishing novel therapeutic pathways for ONP patients with poor prognosis under conventional treatment regimens.

## 2. Case information

The patient and family reported that on August 30, 2024, the patient collided with a small automobile while riding an electric bicycle. Immediately following the accident, the patient lost consciousness and sustained multiple contusions throughout the body. The patient was admitted to the nearest hospital for treatment. Cranial CT revealed bilateral temporal and frontal lobe contusions, subarachnoid hemorrhage, bilateral occipital, and parietal subdural hematomas, pneumocephalus, left occipitoparietal bone fracture, and widening of the left temporo-occipital suture. During hospitalization, the patient received dehydration therapy, phlegm management, and anti-inflammatory treatment. The patient gradually regained consciousness, with normal restoration of independent feeding ability and intestinal function. However, upon awakening, the patient exhibited limited left eye movement and diplopia. Although the patient was discharged after the condition stabilized with subsequent treatment, the limited left eye movement and diplopia symptoms persisted. Consequently, on September 27, 2024, the patient was transferred to our hospital for treatment of TBI and optic nerve pathology.

On admission, the patient underwent a comprehensive rehabilitation assessment, with the initial evaluation focusing on 3 key dimensions: the diplopia scoring method, pupil size contrast, and ocular fissure height contrast (see Table 1). The patient presented with diplopia in most gaze directions, a condition that significantly impaired daily activities and necessitated the occasional covering of 1 eye for functional relief. According to the diplopia scale, this condition was classified as severe diplopia. Regarding ocular fissure height contrast, the left eye exhibited significant ptosis of the upper eyelid, resulting in a 3 mm reduction in the palpebral fissure height. This was deemed to have a moderate impact on daily life and was classified as moderate ptosis. As for pupil size contrast, a difference of 1 to 2 mm was observed, with notable dilation of the left pupil and delayed or diminished light reflexes. This finding was classified as moderate anisocoria.

**Table 1:** Rehabilitation assessment criteria.

Assessment item	Scoring standard	Scoring description
Diplopia scoring method	Grade 0: No diplopia	The patient has no diplopia in any eye position.
	Grade 1: Mild diplopia	Slight diplopia occurs in certain eye positions, which the patient can tolerate and does not affect daily life.
	Grade 2: Moderate diplopia	Double vision is more obvious, usually occurring in multiple eye positions, and the patient needs to adjust their eyes to eliminate the double vision.
	Grade 3: Severe diplopia	Diplopia occurs in most eye positions, severely affecting daily life, and the patient may need to cover 1 eye.
	Grade 4: Extremely severe diplopia	Diplopia is very severe, occurring in all eye positions, and the patient may experience double vision even when the eyes are stationary, severely affecting daily activities.
Pupil Size Comparison	Normal: Both pupils have the same diameter and pupillary reflexes are normal	No anisocoria, and pupillary reflexes are normal.
	Mild anisocoria: Difference <1 mm	In ONP, the affected pupil is slightly larger, and the pupillary reflex is slightly delayed.
	Moderate anisocoria: Difference between 1–2 mm	Due to ONP, the affected pupil is significantly larger, and the reflex is delayed or not obvious.
	Severe anisocoria: Difference >2 mm	The affected pupil is significantly enlarged, and the reflex is absent or extremely sluggish.
Palpebral Fissure Height Comparison	Normal: Both palpebral fissures are of equal height, with no ptosis	No ptosis of the upper eyelid and the palpebral fissures are symmetrical.
	Mild ptosis: Palpebral fissure height difference of 1–2 mm	The upper eyelid of the affected eye is slightly drooping, with minimal impact, usually not affecting daily activities.
	Moderate ptosis: Palpebral fissure height difference of 2–4 mm	The upper eyelid of the affected eye is significantly drooping, reducing the palpebral fissure height and affecting daily life.
	Severe ptosis: Palpebral fissure height difference	The upper eyelid of the affected eye is severely drooping, significantly reducing the palpebral.

## 3. Case analysis

Before considering the cause of motoneural palsy, it is important to understand the anatomical path of the motoneural nerve and the areas where it can be damaged. The motoneural nerve originates in the midbrain and passes through intracranial structures to reach the orbit. Its damage is usually associated with injury or compression of the midbrain and pontine regions.^[17]^ Although CT findings show patchy hyperdense shadows in the temporal and frontal lobes bilaterally, suggesting cerebral contusion, this injury usually does not directly affect the motor neuron unless the midbrain is damaged. Subarachnoid hemorrhage may indirectly affect adjacent brain regions but does not necessarily lead directly to motor nerve palsy. The patient’s imaging showed bilateral subdural hematomas in the occipital and parietal regions and an epidural hematoma in the left parietal region, as well as multiple fractures of the left occipital bone, which may have involved the hindbrain and brainstem but did not directly involve the motor neuron’s area of origin. The most likely contributors to the motor nerve palsy were the left pterygoid fracture and the pterygoid sinus effusion. The area of the pterygoid sinus is a critical part of the motoneural pathway and injury to this area can directly compress or pull on the motoneural nerve, resulting in reduced motoneural function. In addition, a subdural hematoma or increased intracranial pressure may indirectly compress the brainstem region, affecting the motoneural nerve.

**Figure 1.**
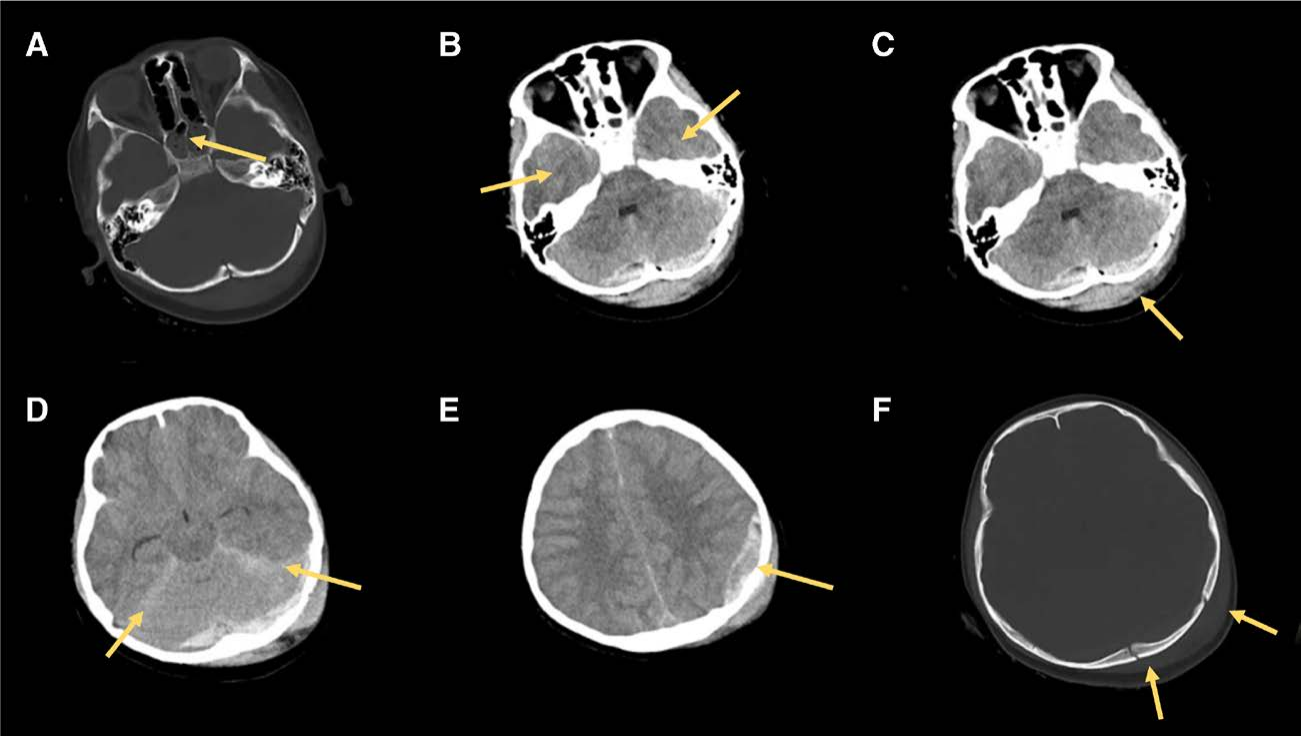
CT scan of the head. (A) Irregularities in the cortex of the right and left lateral walls of the pterygoid mass and the body wall of the pterygoid sinus, consider fracture, pterygoid sinus effusion; (B) mottled hyperdense shadows in the temporal lobes bilaterally; (C) bilateral occipital subdural hematomas, left paraventricular subcortical soft tissue injuries and hematomas; (D) subarachnoid hemorrhage: bilateral occipital subdural hematomas, left parietal occipital subcortical soft tissue injuries and hematoma formation; (E) left parietal epidural hematoma; (F) left occipital multiple fractures, left parietal bone fracture.

The CT scan of the head showed typical manifestations of TBI (Fig. 1), including a left pterygoid fracture, pterygoid sinus effusion, and epidural and subdural hematomas. Imaging studies excluded aneurysms, atherosclerosis, and other vascular lesions that could cause motor nerve palsy. The patient exhibited the classic symptoms of motor nerve palsy, including ptosis, diplopia, unequal pupil size, and limited eye movement. The patient denied a history of diabetes, autoimmune disease, or any other medical condition that could result in neurological dysfunction. After a thorough review of the patient’s medical history, clinical signs, and imaging findings, a diagnosis of “motor nerve palsy caused by craniocerebral injury” was established, ruling out other potential causes of the disease. Therefore, based on the patient’s medical history, clinical manifestations, and imaging findings, the diagnosis of “motor nerve palsy due to craniocerebral injury” was finally confirmed by a comprehensive analysis.

## 4. Treatment

### 4.1. Uyghur medicine treatment

After hospitalization, treatment was administered according to Uyghur Medicine Syndrome Differentiation and Treatment principles, with integrated conditioning implemented based on disease progression. The specific treatment plan includes the use of Fu Fang Ka Sen syrup, it exerts the effects of activating blood circulation, resolving stasis, promoting diuresis, and reducing edema,^[18]^ which may alleviate cerebral edema and facilitate the recovery of brain function. An Shen Gao Zi Ban honey paste^[19]^ primarily functions to calm the mind, nourish the brain, and improve sleep quality, thereby relieving pain and enhancing mental well-being. Qingzhuo Qubiqin Aila honey paste^[20]^ is attributed to clearing heat, eliminating dampness, resolving phlegm, and reducing swelling, potentially aiding in the repair of neural damage. Fu Fang Nu Jia honey paste is believed to tonify the nerves,^[20]^ regulate meridians, and promote blood circulation, offering neuroprotective and regenerative benefits. Fu Fang Ze Hai Pu tablets^[21]^ demonstrate obstruction-clearing and vision-enhancing properties, contributing to improved ocular health. All the above drugs have passed the record and approval of the Provincial Drug Supervision and Administration Department, are produced by our hospital compounding pharmacy, and are limited to Uyghur medical specialties for internal use in our hospital. Details are shown in Table 2.

**Table 2:** Uyghur medicine.

Name of medicine	General name	Usage	Registered number for hospital-prepared formulation
Fu Fang Ka Sen syrup	Compound chicory syrup	20 mL 3 times a day	M20041581
An Shen Gao Zi Ban honey paste	honey paste of the ox-tongue	6 g twice daily	M20041541
Qing Zhuo Qu Bi Qin Ai La honey paste	Poria cocos honey paste	7 g twice daily	M20041572
Fu Fang Nu Jia honey paste	Nujagh honey paste	10 g twice daily	M20041566
Fu Fang Ze Hai Pu tablets	Zahap tablets	5 tablets twice daily	M20041560

### 4.2. Rehabilitation

The rehabilitation protocol was initiated with guided eye movement exercises supervised by a trained therapist. The training regimen commenced with basic cardinal movements (vertical and horizontal gaze) and gradually advanced to incorporate diagonal and oblique movements. During each session, patients were instructed to achieve maximal ocular excursion in all directions, maintaining endpoint fixation for 3 to 5 seconds to enhance extraocular muscle strength and improve neuromuscular coordination, thereby alleviating diplopia. Subsequently, levator palpebrae strengthening exercises were introduced. This involved manual eyelid elevation techniques using digital massage or specialized lid-elevation devices. Patients were instructed to perform active eyelid opening against resistance, maintaining maximum palpebral aperture for sustained periods to improve ptosis. Visual rehabilitation therapy incorporated optical correction with prismatic lenses and occlusive patches. Through systematic visual tracking tasks and pattern recognition exercises, patients were trained to recalibrate visual processing and restore binocular vision. Finally, pupillary reflex training was implemented using graded photic stimulation. Patients were instructed to maintain fixation on variable-intensity light sources, with progressive modulation of luminance to enhance pupillary constriction and dilation dynamics. This comprehensive rehabilitation protocol, characterized by progressive repetition and task-oriented training, was designed to optimize functional recovery. The technical specifications and operational parameters of the oculomotor rehabilitation program are detailed in Table 3.

**Table 3:** Eye movement training.

Training item	Objective	Frequency	Duration
Eye movement exercises	Improve coordination of eye movements	10–15 times/d	20 d
Eyelid lifting training	Enhance eyelid muscle strength	10–20 times/d	20 d
Visual rehabilitation training	Promote visual acuity and reduce diplopia	10–15 times/d	20 d
Pupillary response training	Enhance pupillary sensitivity and accommodation	2–3 times/d	20 d

### 4.3. Acupuncture treatment

Acupuncture treatment aims to cleanse the meridians and harmonize Qi and blood,^[22]^ thereby improving eye movement and enhancing blood circulation, with acupuncture points selected from the Stomach Meridian of Foot-Yangming and the Bladder Meridian of Foot-Taiyang. Primary Acupoints include GV20 (Baihui), EX-HN14 (Eyeming), BL1 (Jingming), LI4 (Hegu), GB37 (Guangming), and Secondary Acupoints include ST36 (Zusanli), SP6 (Sanyinjiao), BL2 (Zanzhu), BL6 (Chengguang), LI14 (Binao), RN6 (Qihai), RN4 (Guanyuan), EX-HN1 (Sishencong), SJ23 (Sizhukong), EX-HN5 (Taiyang), ST1 (Chengqi), GB20 (Fengchi).^[23,24]^ Needling techniques for the eyeball region (ST1, Chengqi) involve the patient closing their eyes, the practitioner gently fixing the eyeball outward and downward, and advancing the needle slowly to a depth of 0.3 cm. For the infraorbital region (BL1, Jingming), the needle is inserted along the infraorbital rim to a depth of 0.3 cm after gently pushing the eyeball upward. TE23 (Sizhukong) is needled obliquely outward and downward to a depth of 0.5 inches. Periocular acupoints (BL1, ST1, TE23) use a scraping and needling technique until Deqi is achieved. LI14 (Binao) is needled obliquely toward the shoulder, quickly piercing 2 to 5 mm under the skin, retained for 3 to 4 seconds, then twisted and manipulated until the needle sensation radiates to the eye. The general needling method for all acupoints involves puncturing perpendicularly with a needle depth ranging from 5 to 70 mm, using the lifting and twisting method, and retaining the needles for 20 minutes. The final step has the patient assume a sitting position, with ST1 (Chengqi) punctured horizontally downward to a depth of 35 mm and GB20 (Fengchi) punctured obliquely upward to a depth of 30 mm, following the direction of the needle.

The following timeline meticulously delineates the dynamic deployment and implementation frequency of various therapeutic components throughout the treatment and rehabilitation process, including Uyghur medicine, rehabilitation training, acupuncture, and monitoring assessment (see Table 4).

**Table 4:** Timeline of comprehensive treatment protocol for oculomotor nerve palsy.

Time point	Uyghur medicine therapy	Rehabilitation training	Acupuncture therapy	Monitoring/assessment
Day 1 (admission)	Initiate all medications: Fu Fang Kasen Syrup (20 mL, 3 times/d). - An Shen Gao Zi Ban honey paste (6 g, 2 times/d) - Qing Zhuo Qu Bi Qin Ai La honey paste (7 g, 2 times/d) - Fu Fang Nu Jia honey paste(10 g, 2 times/d) - Fu Fang Ze Hai Pu Tablets (5 tabs, 2 times/d).	Baseline eye assessments: start basic eye movement exercises (10–15 times/d, guided by therapist).	Initial session: main acupoints (e.g., Baihui, Jingming, Chengqi, Hegu, Guangming); 20–30 min, 2 times/wk.	Baseline: Diplopia scoring, pupil size, palpebral fissure, extraocular muscle activity.
Days 2–9	Continue daily oral administration as above; Adjust dosage if needed based on symptoms	Daily: eye movement (up/down/left/right/diagonal, hold 5–10 s); Eyelid lifting (10–20 times/d); Visual reconstruction training (10–15 times/d)	Weekly sessions (2 times): Auxiliary acupoints (e.g., Zusanli, Sanyinjiao, Yanglao, 3–5 per session); Twisting/lifting-thrusting; Retain needles 10–20 min	Weekly checks: symptom improvement, adverse events.
Day 10 (interim)	Continue medications; Evaluate efficacy (e.g., reduced edema)	Intensify: Add pupil response training (PRT) (2–3 times/d); Focus on coordination	Session with alternated acupoints; Depth 0.3–70 mm	Interim assessment: Scoring updates; Adjust protocol if necessary
Days 11–23	Maintain regimen; Focus on neural nourishment and eye-specific benefits	Daily progression: Increase intensity/duration to max range; 20 d total	Continue 2 times/wk: Emphasize eye-directed needling (e.g., Fengchi to eye, 30 mm)	Bi-weekly monitoring: track recovery metrics
Day 24 (discharge)	Taper or discontinue based on recovery; Provide follow-up prescriptions	Final training session; Home exercises advised	Final session; Evaluate full response	Final assessment: Full recovery confirmed (normal motility, no diplopia, symmetric pupils); SF-36 quality of life score
Post-discharge	The patient took the prescribed discharge medication for 3 wk.	Home-based continuation as needed	Follow-up sessions if required	Long-term follow-up (e.g., 1–3 mo): Relapse monitoring

SF-36 = 36-item Short Form Health Survey.

### 4.4. Ethical statement

The study protocol was approved by the Ethics Committee of Uyghur Medical Hospital of Xinjiang Uyghur Autonomous Region in China (Approval No.: 2025-KY-11), the patient provided written informed consent at the time of admission. The patient was explicitly informed that their clinical data might be used for scientific research and public dissemination, with a strict guarantee to protect patient anonymity and prevent the exposure of personal information or any other data leaks.

## 5. Evaluation of rehabilitation after treatment

### 5.1. Mid-term assessment

After 10 days of treatment, the mid-term rehabilitation assessment revealed significant improvement in the patient’s condition. The patient’s left eye ptosis and left exotropia had completely resolved, with only mild diplopia persisting when gazing inward, which the patient reported as tolerable and not affecting daily activities. Left eye downward, upward, and abduction movements had essentially returned to normal, though adduction movement remained limited. The right pupil dilation had also improved.

### 5.2. Pre-discharge assessment

**Figure 2.**
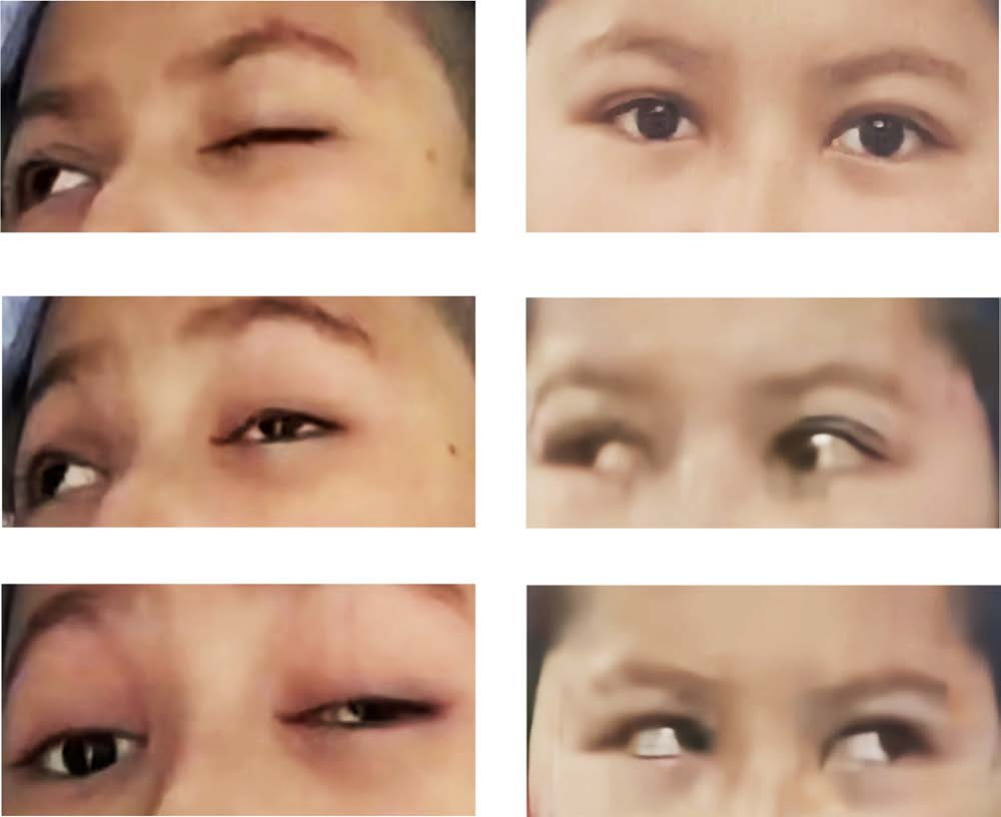
Comparison of before and after rehabilitation.

After 24 days of treatment, the patient underwent another rehabilitation assessment prior to discharge (see Fig. 2). Assessment results demonstrated complete resolution of left eyelid ptosis, left eye strabismus, and diplopia symptoms, which no longer affected daily functioning. Left eye movements in all directions (upward, downward, inward, and outward) had essentially returned to normal, and bilateral pupils were symmetrical in size with regular morphology. Comparative analysis of rehabilitation assessment data at admission, after 10 days of treatment, and after 24 days of treatment (pre-discharge) is detailed in Table 5.

**Table 5:** Comparison of assessment items.

Assessment item	Admission (baseline)	After 10 d of treatment (October 7, 2024)	Before discharge (after 24 d of treatment, October 21, 2024)	Improvement notes
Diplopia scoring	Level 3: severe diplopia (occurs in most gaze positions, severely affects daily life, may require eye patching).	Level 1: mild diplopia (slight when gazing inward, tolerable, does not affect daily life).	Level 0: no diplopia (absent in all gaze positions, does not affect daily life).	Progressed from severe to none, indicating full recovery of ocular coordination, possibly related to acupuncture regulating BDNF expression and reducing inflammation.
Pupil size comparison	Moderate anisocoria: Difference 1–2 mm (left pupil significantly dilated, reflex delayed or absent).	Moderate anisocoria: difference approximately 1 mm (improved, but reflex still delayed).	Normal: no difference (bilateral pupils equal in size, reflex normal).	Progressed from moderate anisocoria to normal, with difference eliminated, suggesting pupil function regeneration possibly via improved local blood flow.
Palpebral fissure height comparison	Moderate ptosis: palpebral fissure height difference 3 mm (obvious left upper eyelid droop, affects daily life).	Normal: bilateral palpebral fissure heights equal, no ptosis.	Normal: bilateral palpebral fissure heights equal, no ptosis (upper eyelid normal).	Rapid recovery from moderate ptosis to normal, indicating significant improvement in levator palpebrae superioris function, related to acupuncture promoting nerve regeneration.
Extraocular Muscle Activity Score (Supplementary, based on journal suggestion; 0 = normal, 4 = complete paralysis).	Level 3: severe paralysis (extraocular muscle activity severely restricted).	Level 2: moderate paralysis (downward/upward/outward movements basically normal, inward movement still restricted).	Level 0: normal (downward/upward/inward/outward movements basically restored to normal).	Improved from severe to normal in all directions, showing significant overall functional progress, possibly requiring further validation in Figure 2.

BDNF = brain-derived neurotrophic factor.

### 5.3. 36-item Short Form Health Survey assessment of the quality of life

To evaluate the impact of ONP on the patient’s quality of life and treatment effectiveness, the 36-item Short Form Health Survey was administered alongside standard rehabilitation assessments. Baseline evaluation revealed impairment across all dimensions. Following treatment implementation, significant improvements were documented at both the 10-day mark and pre-discharge assessment. In the pre-discharge assessment, the patient reported that their quality of life had nearly completely recovered, with scores exceeding 90 points in most physical and social dimensions, indicating good overall health status. All evaluations were conducted by rehabilitation therapist with intermediate or higher professional credentials, ensuring assessment reliability (see Table 6).

**Table 6:** Comparison of SF-36 assessment items.

SF-36 domain	At admission (day 1)	After 10 d of treatment	Before discharge (day 24)
Physical functioning (PF)	45	75	95
Role-physical (RP)	20	60	90
Bodily pain (BP)	50	75	90
General health (GH)	45	65	80
Vitality (VT)	30	60	85
Social functioning (SF)	25	70	95
Role-emotional (RE)	30	65	90
Mental health (MH)	40	70	85

SF-36 = 36-item Short Form Health Survey.

## 6. Follow-up records

**Figure 3.**
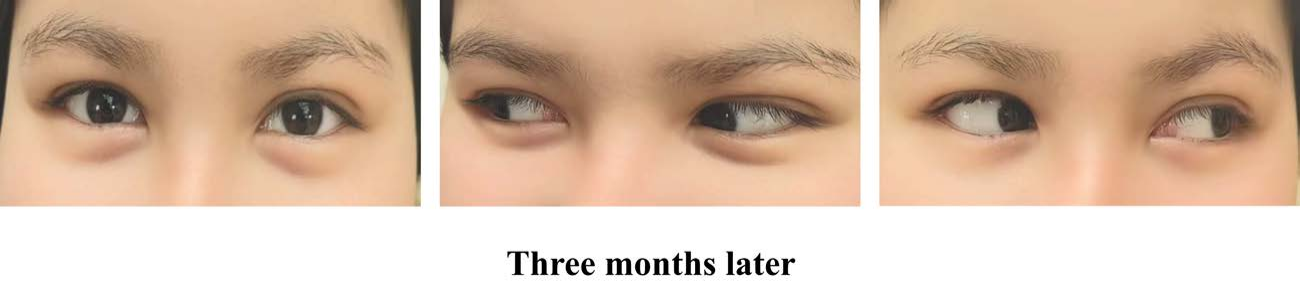
Follow-up pictures.

During the 3-month follow-up period after discharge, the patient reported no new symptoms or complaints. Physical examination revealed no ptosis of the left eyelid, with free movement of the eyeball; bilateral pupils were symmetrical in size with regular morphology and normal light reflexes (see Fig. 3). The patient reported no impact on daily activities and an improvement in quality of life.

**Figure 4.**
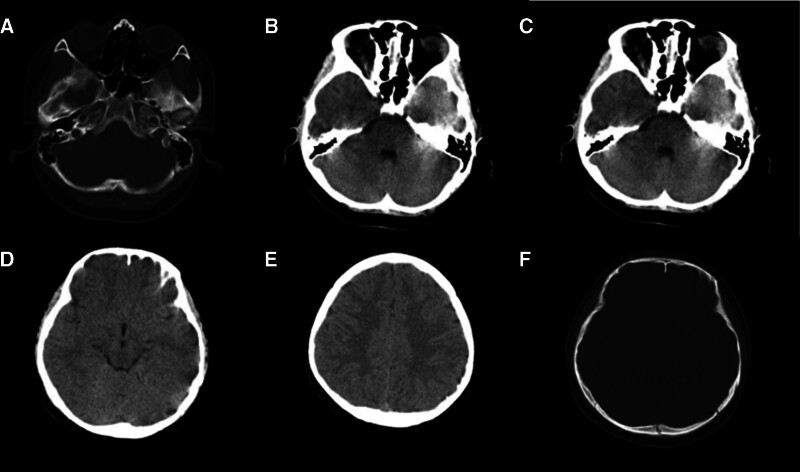
Follow-up CT image. (A) Sphenoid sinus: irregularities in the cortical bone of the bilateral lateral walls and body of the sphenoid sinus (now consistent with old fractures). Resolution of sphenoid sinus fluid accumulation (previously described as sinus effusion); (B) bilateral temporal lobes: previously observed patchy high-density shadows (suggestive of cerebral contusion/laceration) have resolved completely, with no residual abnormalities detected; (C) hemorrhage evolution: bilateral occipital subdural hematomas: completely absorbed. Left occipitoparietal subcutaneous soft tissue injury and hematoma: fully resolved; (D) subarachnoid hemorrhage: complete resolution observed, with no evidence of residual bleeding; (E) left parietal epidural hematoma: absorbed without sequelae; (F) fracture status: left occipital bone (multiple fractures) and left parietal bone fracture: healed as old fractures with cortical remodeling. New finding: mild widening of the left temporo-occipital suture, suggesting possible subtle diastasis (clinical correlation advised).

Follow-up cranial CT showed multiple nodular high-density shadows visible below the left parietal region and an old occipital bone fracture. Compared with the previous images from September 18, 2024, there was no evidence of bilateral frontal and temporal lobe contusions; the subarachnoid hemorrhage, bilateral occipital and parietal subdural hematomas, and left epidural hematoma had been completely absorbed (see Fig. 4).

## 7. Discussions

This report describes a case of ONP caused by craniocerebral injury. The patient received a comprehensive treatment plan combining oral Uyghur medicine, acupuncture, and rehabilitation training. After 24 days of treatment, the patient achieved complete recovery: left eye movements returned to normal, symptoms of diplopia and ptosis disappeared, and pupillary light reflex normalized. This case suggests that the integration of Uyghur medicine with acupuncture and rehabilitation training can be an effective strategy for treating refractory conditions such as ONP.

Uyghur medicine follows the principles of holistic and radical treatment in treating diseases.^[25]^ For craniocerebral injury and cerebral edema, Fu Fang Ka Sen syrup, with chicory as its main ingredient, was administered. It is traditionally used for its diuretic and anti-swelling effects.^[26]^ Studies show chicory’s active components can modulate oxidative stress and suppress inflammatory responses, thereby ameliorating tissue edema.^[27]^ Clinically, the patient reported a significant increase in urination without signs of electrolyte imbalance, demonstrating chicory’s diuretic and anti-edematous properties which are crucial for relieving intracranial pressure in a TBI case. An’shen Gaoziban honey paste is prescribed for neuroprotective and sedative purposes. Its primary active component, *Anchusa italica* (commonly known as ox-tongue or bugloss), has demonstrated significant anti-inflammatory, antioxidant, and neuroprotective properties.^[28]^ TBI involves multiple pathological mechanisms, including excitotoxicity and cerebral edema.^[29]^ The patient reported notable improvement in headaches, restlessness, and insomnia, which aligns with studies showing *A italica* extract mitigates free radical damage and inflammatory responses, potentially by regulating brain-derived neurotrophic factor mRNA.^[30]^ Fu Fang Nu Jia honey paste was also used for its neuroprotective effects. Its extract has been shown to alleviate neural damage, likely through the activation of anti-inflammatory and antioxidant pathways, offering therapeutic benefits for neural tissue injury.^[31]^ Qing Zhuo Qu Bi Qin Ai La honey paste is known to reduce abnormal body fluids and possesses anti-inflammatory, anti-edema, and analgesic effects.^[32]^ Studies have confirmed its ability to reduce inflammatory cytokines such as IL-6, IL-8, and TNF-α.^[33]^ Its application was intended to reduce local inflammation and edema around the affected nerve, a key factor in nerve compression and dysfunction. Fu Fang Ze Hai Pu tablets were administered for the purpose of brightening and benefiting the eyes which included in the ministerial standard “Chinese medicine prescription preparation Uyghur medicine subsection” (WS3-BW-0173-98), formulation is composed of medicinal ingredients such as the flesh of *Terminalia chebula* (Chebulic Myrobalan), *Convolvulus scammonia* resin (Scammony), *Aloe vera* (Aloe), and *Crocus sativus* (Saffron). Its primary efficacy lies in its remarkable ability to remove visual obstructions and improve eyesight, highlighting its potential as a therapeutic agent for ocular disorders.^[34]^ The patient reported significant relief from eye pressure, intermittent blurred vision, and headaches after taking the tablets. Its primary constituents, aloe vera and saffron, are known to possess neuroprotective properties by modulating apoptosis, inflammation, and oxidative stress.^[31,35]^

Oculomotor training constitutes a fundamental element of the rehabilitation program, intending to enhance eye muscle strength and coordination, whilst concomitantly reducing diplopia symptoms. Eye movement training has been demonstrated to result in a substantial augmentation of the range and velocity of eye movements in patients afflicted with motor nerve palsy. Furthermore, the execution of diagonally oriented movements has been shown to facilitate the activation of the ocular muscles to a greater extent, thereby amplifying the therapeutic effect. Eyelid lift training is imperative in the management of ptosis symptoms. Eyelid lift training enhances eyelid function by increasing the strength and endurance of the eyelid muscles, facilitating the movement of closing the eyes and then forcefully opening them, which may help to increase the responsiveness and coordination of the eyelid muscles. Visual reconstruction training assists patients in adapting to new visual information through the use of corrective glasses and prisms or eye shields, promoting the brain’s reintegration of visual information and enhancing visual acuity. Pupil response training involves the stimulation of the pupil with different light intensity levels to enhance sensitivity and adjust to light. Pupil response training can improve the contraction and dilation of the pupil, facilitating adaptation to different light environments.

Acupuncture therapy constitutes a primary therapeutic modality within the framework of TCM. Recent research has demonstrated that acupuncture promotes the release of brain-derived neurotrophic factor and vascular endothelial growth factor.^[36]^ These molecules function as crucial trophic mediators, promoting the survival of neural stem cells and stimulating the growth of new nerves and the migration of neuronal generative regions.^[37]^ Recent the other studies shows that acupuncture on periocular acupoints can elevate mean blood flow in arteries and promote local hcapillary anastomosis and fusion.^[38]^ Acupuncture treatment has been shown to reduce angiotensin II levels in the blood and increase the bioavailability of nitric oxide, leading to capillary dilatation and enhanced reperfusion, and thus improving neurological function after microvascular brain tissue injury.^[39]^ Furthermore, it has been demonstrated that the needling of specific acupoints, such as Wei Guan, can stimulate brain regions associated with vision, enhance cerebral cortex blood flow, and promote the recovery of nerves in damaged brain areas.^[40]^ Several case studies have demonstrated the significant efficacy of acupuncture in the treatment of ONP.^[41]^ For instance, a meta-analysis of acupuncture for the treatment of ONP summarized 18 randomized controlled trials (RCTs) involving 1150 participants, and the combined report suggested that acupuncture does have certain efficacy in improving ONP. However, these studies were predominantly conducted in China, and the overall quality of the evidence is limited.^[42]^ Theoretically, acupuncture is a rational treatment for motor nerve palsy post-craniocerebral injury by creating favorable conditions for nerve repair through neuro-modulatory, vascular, and anti-inflammatory effects. However, substantiating this therapeutic rationale requires more definitive, high-quality clinical evidence.

Repeated cranial CTs revealed that the resolution of epidural and subdural hematomas provided the necessary space for neurological recovery, likely by alleviating compression on the brainstem and motoneuron pathways. Concurrently, the healing of the pterygoid sinus fracture may have reduced direct mechanical and inflammatory stress on the oculomotor nerve.Recovery is likely attributable to both the repair of the primary injury (e.g., axonal regeneration) and central compensatory mechanisms, such as brainstem functional reorganization. Adjuvant therapies, including acupuncture and rehabilitation, may have potentiated this process by enhancing microcirculation, reducing edema, and facilitating systematic retraining of the ocular-motor system.

Although the multimodal approach combining Uyghur medicine, rehabilitation, and acupuncture showed significant therapeutic effects in this case, the synergy between these therapies remains speculative. Postulated mechanisms include Uyghur medicine’s anti-inflammatory properties enhancing acupuncture’s neuro-regenerative effects, and rehabilitation amplifying acupuncture’s impact on local microcirculation. This aligns with multimodal treatment theories for TBI, but such synergistic inferences are based on preclinical data and case observations, not high-level evidence. Consequently, this study highlights a potential (but unconfirmed) synergy. Future RCTs are imperative to validate these interaction effects and move beyond theoretical postulation.

The primary limitation of this study is its single-case design, which prohibits generalizability and is susceptible to selection bias from unique patient-specific factors. The findings are further constrained by a reliance on subjective clinical assessments without objective neurophysiological data (e.g., EMG), introducing a risk of observer bias. The absence of a control group also means that the observed improvements cannot be definitively attributed to the intervention over spontaneous recovery or other confounders. The brief 3-month follow-up may also be insufficient to evaluate long-term efficacy. Therefore, this report serves primarily to generate a hypothesis for future investigation. A staged approach is required to validate these findings. Initially, a prospective cohort study is warranted to assess preliminary efficacy and safety in a broader patient sample. Positive outcomes would then justify a more rigorous single-center RCT to compare the integrated therapy against a standard-of-care control. Ultimately, if efficacy is confirmed, a large-scale, multicenter RCT would be essential to provide definitive, high-level evidence and establish this multimodal approach as an evidence-based therapy for traumatic ONP.

## Author contributions

**Data curation:** Nilupaer Xiefukaiti, Yang Liu, Gulinazi Rouzi, Aerziguli Aizezi.

**Investigation:** Gulinishahan Abulimiti, Nilupaer Xiefukaiti, Yang Liu, Gulinazi Rouzi, Aerziguli Aizezi, Zehuai Wen.

**Methodology:** Nilupaer Xiefukaiti, Aerziguli Aizezi, Zehuai Wen.

**Supervision:** Aizezi Aihemaitiniyazi.

**Validation:** Aizezi Aihemaitiniyazi.

**Writing – original draft:** Ailiyaer Yasheng, Gulinishahan Abulimiti, Aizezi Aihemaitiniyazi.

**Writing – review & editing:** Ailiyaer Yasheng, Aizezi Aihemaitiniyazi.
